# Successful treatment of relapsed and refractory CIDP with ofatumumab: a first case report

**DOI:** 10.3389/fimmu.2024.1437848

**Published:** 2024-07-31

**Authors:** Jian Wang, QunHong Xiang

**Affiliations:** ^1^ Department of Neurology, Affiliated Aerospace Hospital of Zunyi Medical University, Zunyi, Guizhou, China; ^2^ Department of Neurology, People’s Hospital of Wuchuan County, Zunyi, Guizhou, China

**Keywords:** chronic inflammatory demyelinating polyradiculoneuropathy, relapse, refractory, ofatumumab, efficacy

## Abstract

**Background:**

Chronic inflammatory demyelinating polyradiculoneuropathy (CIDP) is a heterogeneous but treatable immune-mediated neuropathy. Ofatumumab (OFA) is a fully human anti-CD20 monoclonal antibody that has shown promising efficacy in central demyelinating diseases, such as multiple sclerosis (MS). However, there is a lack of studies on the usage of OFA in peripheral demyelinating diseases, particularly CIDP. A case of relapsed and refractory CIDP with an ineffective response to conventional immunotherapy and intolerance to rituximab (RTX) but a positive response to subcutaneous injections of OFA is presented.

**Case presentation:**

The patient, a 46-year-old man diagnosed with CIDP, received high-dose intravenous methylprednisolone, intravenous immunoglobulin (IVIG), and plasma exchange(PE) during the acute phase of the disease, and long-term oral administration of prednisone, azathioprine (AZA), and mycophenolate mofetil (MMF) during the remission phase. However, the patient suffered six relapses over a five-year period, and because of these, along with an ineffective response to conventional immunotherapy, and intolerance to RTX, subcutaneous injections of OFA were selected as a prophylactic treatment against relapses. After a total of six injections of OFA, CD19+B cells were substantially depleted. The patient has been followed for more than 23 months without relapse.

**Conclusions:**

This case demonstrates the effectiveness and good tolerability of OFA in the treatment of relapsed and refractory CIDP. Further studies are needed to investigate the efficacy and safety of OFA in patients with relapsed and refractory CIDP, especially in those who have shown an ineffective response to conventional immunotherapy and are intolerant to RTX.

## Introduction

Chronic inflammatory demyelinating polyradiculoneuropathy(CIDP) is a type of immune-mediated peripheral neuropathy characterized by bilateral symmetrical paralysis and numbness in the limbs, sensory disturbances in the extremities, reduced or absent reflexes in the limbs, and a disease duration exceeding 8 weeks (1). Electrodiagnostic criteria demonstrated demyelination-related changes, including reduced conduction velocity, prolonged distal latency, motor nerve conduction block, abnormal waveform dispersion and undetectable F-wave. The majority of patients showed significant functional improvement following immunotherapy. However, a minority experience a prolonged disease course necessitating long-term drug maintenance while others demonstrate a negative response to treatment resulting in severe neurological disability (1, 2). Therefore, for patients who are experiencing frequent relapses or ineffective responses to conventional immunotherapy, there is a need for more effective biological agents such as Ofatumumab (OFA), which is a fully human anti-CD20 monoclonal antibody that has obtained approval for the modifying treatment of MS (3). Studies have shown that OFA has promising efficacy as a salvage or first-line treatment in MS (3, 4). However, there is a gap in the literature regarding its use in peripheral demyelinating diseases, particularly in relapsed and refractory CIDP. A case is presented of CIDP characterized by frequent relapses, an ineffective response to conventional immunotherapy, and intolerance to RTX. The patient demonstrated a good response to a subcutaneous injection of OFA without any complications.

## Case report

The patient, a 46-year-old man, had been complaining of limb weakness for approximately three months. According to the patient’s description, in Feb.2017, he experienced limb weakness without any apparent cause. This was characterized by an inability to grasp objects with both hands, weakness in both lower limbs, and a loss of independent walking ability; the patient typically relied on a wheelchair for outdoor travel but could manage to stand and take a few steps with the assistance of family members. No symptoms such as headache, vomiting, blurred vision, incontinence, dyspnea or unconsciousness were observed during the course of the disease. The patient was admitted to our department for inpatient care. A physical examination showed proximal strength grade 4 and muscle distal strength grade 2 of both upper and lower limbs, poor grip strength of both hands, absence of bilateral biceps and triceps reflexes, as well as patellar and ankle reflexes, and gloves -socks senory loss hypoesthesia in the distal limbs. The inflammatory neuropathy cause and treatment disability score(INCAT) was 6 points, the cerebrospinal fluid (CSF) examination revealed normal intracranial pressure and cell count in addition to a significant increase in CSF-protein, and the electrophysiological examination revealed a reduction in conduction velocity of the bilateral median, ulnar, common peroneal, and tibial nerves. In addition, there was a significant lengthening of delay and a significant reduction of amplitude with dispersion, with an F-wave abnormality observed in both the bilateral median and right tibial nerves, whereas the F-wave was not detectable in the bilateral ulnar and left tibial nerves. An MRI of the brain and spinal cord revealed no abnormalities, while the levels of ganglioside antibodies were within the normal range. The patient was diagnosed with CIDP. The patient’s limb weakness gradually returned to normal after receiving intravenous immunoglobulin(IVIg dose 0.4g/kg/day) for five consecutive days. Since his discharge, he has regained Grade 5 muscle strength in all his limbs. Oral prednisone was then administered at a maintenance dose of 30 mg. In Jan.2018, the patient was readmitted to the hospital due to limb weakness and numbness without inducement. A physical examination revealed grade 3 proximal and grade 1 distal muscle strength in both upper and lower limbs, absence of limb tendon reflexes, and glove stock-like hypoesthesia in the distal limbs. The INCAT score was 7. The patient was diagnosed with relapsed CIDP. We administered IVIg therapy (0.4g/kg/day) for five consecutive days, along with methylprednisolone treatment (1000mg ivgtt for 3 days, 500mg ivgtt for 2 days). The patient’s paralysis gradually improved. After discharge, the patient was advised to begin oral prednisone at a dose of 30 mg, to be tapered slowly, and continued for six months. Unfortunately, in October 2018, the patient was admitted to our department due to limb paralysis with no apparent cause. A physical examination revealed grade 3 proximal and grade 2 distal muscle strength, absence of limb tendon reflexes, glove-like hypoesthesia in the distal limb, and an INCAT score of 6. A diagnosis of CIDP relapse was made. We administered IVIg therapy (0.4g/kg/day) for five consecutive days, along with methylprednisolone treatment (1000mg ivgtt for 3 days, 500mg ivgtt for 2 days). The patient underwent rehabilitation, and the paralysis gradually improved. Upon discharge, the patient was treated with low-dose prednisone for six months, and monthly intravenous immunoglobulin for five consecutive days. The patient underwent three cycles of treatment due to high medical costs.

In Jul. 2019, the patient suffered a third relapse and was admitted to the hospital due to limb paralysis. A physical examination revealed grade 3 proximal and grade 2 distal muscle strength, absence of limb tendon reflexes, and gloves -socks senory loss hypoesthesia in the distal limbs. The INCAT score was 6, and he was diagnosed with relapsed CIDP. The patient received IVIg therapy (0.4g/kg/day) for five consecutive days, in combination with methylprednisolone treatment (1000mg ivgtt for 3 days, 500mg ivgtt for 2 days). Additionally, the patient underwent rehabilitation, and the limb paralysis gradually improved. Subsequently, the patient was treated with IVIg, which was administered once a month for a total of three cycles, each lasting five days. Due to the patient’s frequent relapses, we made adjustments to the immunotherapy regimen by reducing the prednisone dosage to 15mg, along with maintenance therapy of azathioprine (AZA 50mg bid). Unfortunately, in Apr. 2020, the patient suffered a fourth relapse and was admitted to the hospital due to paralysis and numbness in the limbs. A physical examination revealed grade 4 proximal and grade 2 distal muscle strength, absence of limb tendon reflexes, and Gloves -socks senory loss hypoesthesia. The INCAT score was 6, and the patient was diagnosed with relapsed CIDP. He received five time of plasma exchange therapy(PE 40ml/kg/time), and the limb paralysis gradually improved. Due to the high medical costs, the patient received only two cycles of treatment. We made further adjustments to the immunotherapy regimen by discontinuing oral AZA and adding mycophenolate mofetil (MMF,1000mg tid), along with low-dose prednisone (15mg qd) for maintenance therapy.

In Mar. 2021, the patient suffered a fifth relapse and was admitted to the West China Hospital of Sichuan University due to limb paralysis. The INCAT score was 7, and the patient was diagnosed with relapsed CIDP. The serum tests for paraneoplastic syndrome, gangliosides, paranodal and autoimmune nodes of Ranvier antibodies were all within the normal range. The patient received IVIg therapy (0.4g/kg/day) for five consecutive days, in combination with methylprednisolone treatment (1000mg ivgtt for 3 days, 500mg ivgtt for 2 days). In addition, the patient’s limb paralysis gradually improved with rehabilitation. He then received immunoglobulin therapy once a month for a total of four cycles, each lasting five consecutive days. Upon discharge, he was placed on a combination therapy of MMF(1000mg tid) and prednisone (15mg qd). The patient remained stable for approximately 12 months.

**Figure 1:**
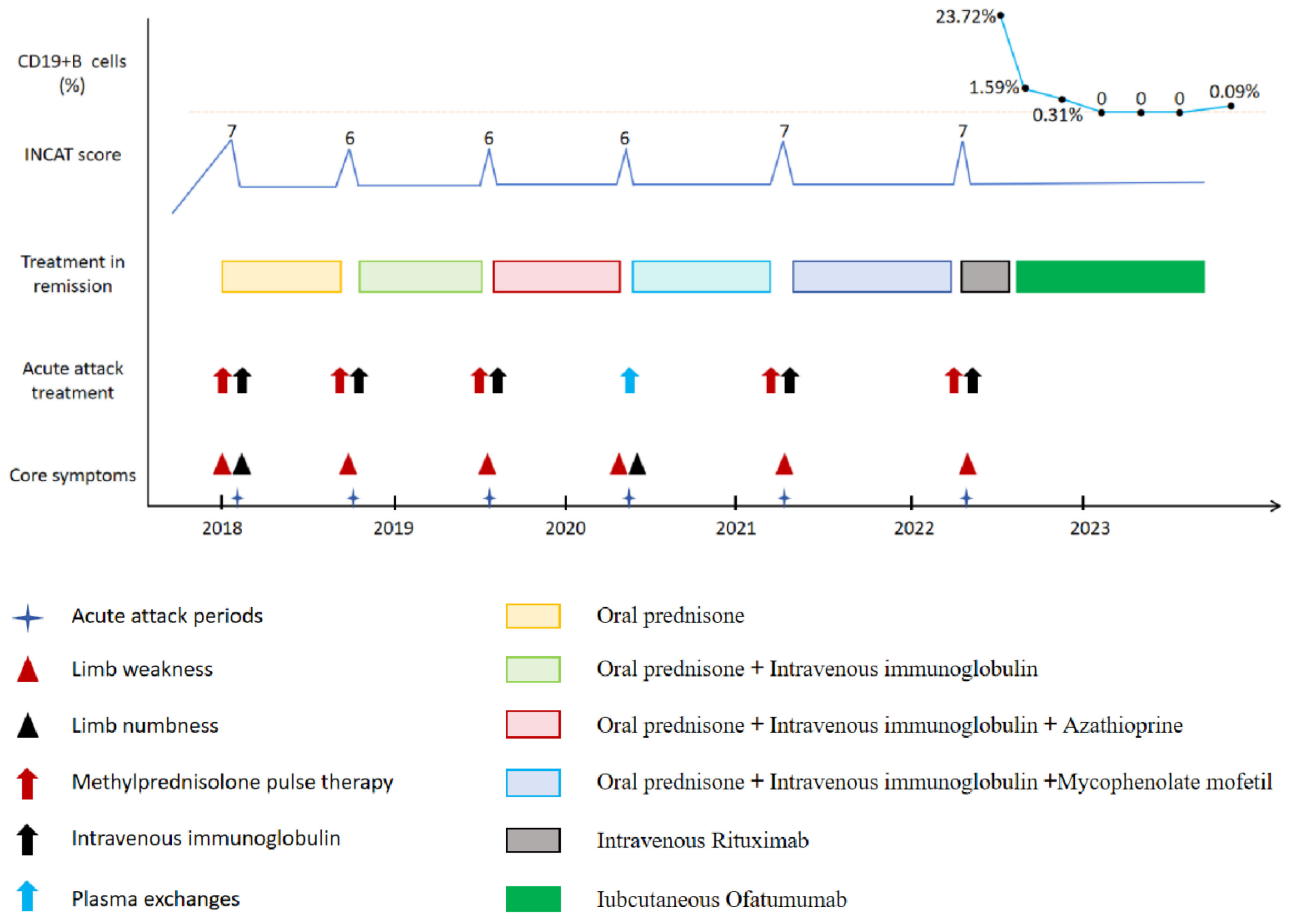
Schematic illustration of disease progression showing relapse frequency and core symptoms, severity of disability, treatment regimens, and CD19+B cell levels.

In Apr. 2022, the patient had a sixth relapse and was admitted to the hospital with limb paralysis. A physical examination revealed grade 3 proximal and grade 1 distal muscle strength. Additionally, there was an absence of limb tendon reflexes and a shallow sensory loss resembling a glove or sock in the distal limbs. The INCAT score was 7. The patient was diagnosed with relapse CIDP. The results of serum and urine immunofixation electrophoresis, free light chain tests, and bone marrow aspiration were within normal limits. We were given consecutive five time IVIg(0.4g/kg/day), in combination with methylprednisolone (1000mg ivgtt 3 days, 500mg ivgtt 2 days) therapy, and his limb paralysis has gradually improved. The patient’s detailed 5-year medical history was reviewed because of multiple relapses. Throughout the maintenance treatment phase, the patient consistently adhered to the medication without any interruptions, and no discernible adverse drug reactions were observed to cause the patient’s frequent relapses, multiple traditional immunotherapy regimens ineffectively prevent disease. We provided the patient with a comprehensive explanation of the condition and administered an intravenous infusion of RTX (first dose 1000mg). However, approximately half an hour after the infusion, the patient suddenly developed shortness of breath, severe cough, and enormous urticaria on the chest and back. He was immediately given Intravenous dexamethasone and calcium gluconate therapy. The symptoms gradually improved. On the second day after RTX treatment, he presented with a productive cough accompanied by white mucous sputum and pyrexia, with a maximum temperature of 39°C, positive streptococcus pneumoniae growth in the sputum bacterial culture, and pulmonary infection as suggested by a lung CT examination. The patient received continuous intravenous administration of Cefazolin (2g ivgtt q12h) for a duration of two weeks, after which he recovered from a pulmonary infection. Due to the adverse drug effects, subsequent RTX therapy was refused by the patient. Given his frequent relapses, inadequate response to conventional immunotherapy, and intolerance to RTX treatment, we reiterated the condition to the patient and recommended OFA as a preventive measure against relapses. After obtaining the patient’s informed consent, subcutaneous administration of OFA was initiated on 29 Apr. 2022 (20mg on days 1, 7, and 14, followed by a monthly dose of 20mg). Throughout the treatment course no adverse drug reactions were observed, and following a total of six injections, the depletion of CD19+B cells was essentially complete. The patient has been followed for more than 23 months and has not relapsed (**Figure 1**).

## Discussion

CIDP is an immune-mediated autoimmune peripheral demyelinating disease that has a prevalence of 0.67-10.3 cases per 100,000 population and an incidence of 0.15-10.6 cases per 100,000 population. These rates demonstrate a positive correlation with age; the condition is more commonly observed in men (1). The chronic, progressive or relapsing nature of CIDP manifests itself in a multifocal manner, affecting spinal nerve roots, plexuses, and nerve trunks to varying degrees. Partial or complete recovery between relapses is observed, with a recurring course typically seen in younger patients and a progressive course more commonly observed in older patients (5). Dysfunction or absence of immune tolerance mechanisms (6, 7) is considered to be the common immunological basis of CIDP and MS, and it plays a crucial role in CIDP and MS, which can cause autoimmune responses in myelin and axonal complexes.

The majority of cases of CIDP respond appropriately to glucocorticoid therapy, but a small percentage do not show sensitivity to glucocorticoids or conventional immunotherapy. In such cases, extremely effective biological agents like B cell-depleting anti-CD20 monoclonal antibodies are required (8). The most commonly used human-mouse chimeric anti-CD20 monoclonal antibody is RTX, which has been clinically utilized for the disease-modifying treatment of various autoimmune and demyelinating diseases, such as MS (9). Studies (9, 10) have shown that while RTX has favorable efficacy in preventing relapses of autoimmune and demyelinating diseases, its clinical application may be limited by severe allergic reactions, the generation of antidrug antibodies, and an elevated susceptibility to infection. OFA is a fully humanized anti-CD20 monoclonal antibody that, in contrast to the epitopes recognized by RTX, exerts its effect by binding to two distinct epitopes, thereby inducing complement-mediated CD20+B-cell lysis and antibody-dependent cell-mediated cytotoxicity for effective elimination of pathogenic B cells (11). A fully human design significantly reduces the likelihood of treatment failure by avoiding the risk of developing anti-drug antibodies, and injection-related systemic reactions are fewer than those associated with the infusion. In addition, drug administration is convenient and can be performed by the patients themselves (12). In our case, the use of RTX resulted in severe infusion reactions and infections. Fortunately, OFA was well-tolerated by the patient, with no adverse drug effects. Moreover, it effectively depletes circulating CD19+B cells. Nonetheless, this patient has not been on OFA long enough to observe the long-term efficacy and safety of OFA in the treatment of relapsed and refractory CIDP.

In conclusion, the first use of subcutaneous injection of OFA in relapsed and refractory CIDP patients presented here suggests that OFA may be a well-tolerated and effective alternative. Further studies are needed to investigate the efficacy and safety of OFA in patients with relapsed and refractory CIDP, especially in those who have had an ineffective response to conventional immunotherapy or who are intolerant to RTX.

## Data availability statement

The datasets presented in this article are not readily available because of ethical and privacy restrictions. Requests to access the datasets should be directed to the corresponding author.

## Ethics statement

The studies involving humans were approved by the Affiliated Aerospace Hospital of Zunyi Medical University. The studies were conducted in accordance with the local legislation and institutional requirements. The participants provided their written informed consent to participate in this study. Written informed consent was obtained from the individual(s) for the publication of any potentially identifiable images or data included in this article.

## Author contributions

JW: Writing – review & editing, Writing – original draft, Visualization, Validation, Supervision, Software, Resources, Project administration, Methodology, Investigation, Funding acquisition, Formal analysis, Data curation, Conceptualization. QX: Writing – original draft, Visualization, Supervision, Resources, Project administration, Methodology, Investigation, Data curation.
