# Supplementary material for: Successful treatment of relapsed and refractory CIDP with ofatumumab: a first case report
Source: Front Immunol. 2024 Jul 31;15:1437848. doi: 10.3389/fimmu.2024.1437848 (PMC11323304; doi:10.3389/fimmu.2024.1437848)
Supplement: Supplementary file 1 [file DataSheet_1.pdf]

Partial electroneuromyography of the patient

Sensory nerve conduction

感觉传导 SNC

Annotation	神经 / 部位	Onset Lat ms	Peak Lat ms	NP Amp μV	PP Amp μV	段 (position)
Left median nerve	L 正中神经 - Digii II Antidromic)					
	Wrist	0.00	0.00	0.00	0.00	Wrist - DigII
Right median nerve	R 正中神经 - Digii II Antidromic)					
	Wrist	0.00	0.00	0.00	0.00	Wrist - DigII
Left ulnar nerve	L 尺神经 - Digit V (Antidromic)					
	Wrist	0.00	0.00	0.00	0.00	Wrist - Dig V
						A.Elbow - Wrist
Right ulnar nerve	R 尺神经 - Digit V (Antidromic)					
	Wrist	0.00	0.00	0.00	0.00	Wrist - Dig V
						A.Elbow - Wrist
Right sural nerve	R 腓肠神经 - Ankle (Calf)					
	Calf	0.00	0.00	0.00	0.00	Calf - Ankle
Left sural nerve	L 腓肠神经 - Ankle (Calf)					
	Calf	0.00	0.00	0.00	0.00	Calf - Ankle
Right superficial peroneal nerve	R 腓浅神经 - Ankle					
	Lat leg	0.00	0.00	0.00	0.00	Lat leg - Ankle
Left superficial peroneal nerve	L 腓浅神经 - Ankle					
	Lat leg	0.05	0.05	0.00	0.00	Lat leg - Ankle

Motor nerve conduction

运动传导 MNC

Annotation	神经 / 部位	Muscle	Latency ms	Amplitude mV	Rel Amp %	Duration ms	段 (position)	Distance mm	Lat Diff ms	Velocity m/s
Left median nerve	L 正中神经 - APB									
	Wrist		10.52	4.4	100	10.89	Wrist - G1			
	B.Elbow		18.54	1.5	34.4	14.43	B.Elbow - Wrist	210	8.02	26
Right median nerve	R 正中神经 - APB									
	Wrist		11.82	4.2	100	9.74	Wrist - G1			
	B.Elbow		23.18	0.9	20.6	8.59	B.Elbow - Wrist	260	11.35	23
Left ulnar nerve	L 尺神经 - ADM									
	Wrist	ADM	11.61	0.7	100	11.41	Wrist - ADM			
	B.Elbow	ADM	21.25	1.0	147	11.25	B.Elbow - Wrist	260	9.64	27
							A.Elbow - Wrist			
Right ulnar nerve	R 尺神经 - ADM									
	Wrist	ADM	12.08	0.4	100	7.71	Wrist - ADM			
	B.Elbow	ADM	25.05	1.3	379	9.95	B.Elbow - Wrist	260	12.97	20
							A.Elbow - Wrist			
Right tibial nerve	R 胫神经 - AH									
	Ankle	AH	14.38	0.6	100	8.80	Ankle - AH			
	Pop fossa	AH	32.71	0.2	26.7	5.21	Pop fossa - Ankle	320	18.33	17
Left tibial nerve	L 胫神经 - AH									
	Ankle	AH	15.83	1.4	100	8.70	Ankle - AH			
	Pop fossa	AH	26.56	0.5	38.7	8.91	Pop fossa - Ankle	320	10.73	30
Right common peroneal nerve	R 腓总神经 - EDB									
	Ankle		11.93	2.1	100	7.92	Ankle - G1	80		
	Below Fiblula		0.00	0.0	0	0.00	Below Fiblula - Ankle		-11.93	0

Annotation	神经 / 部位	Muscle	Latency ms	Amplitude mV	Rel Amp %	Duration ms	段	Distance mm	Lat Diff ms	Velocity m/s
Left common peroneal nerve	L 腓总神经 - EDB									
	Ankle		5.89	1.2	100	12.66	Ankle - G1			
	Below Fiblula		20.52	0.4	38.2	5.31	Below Fiblula - Ankle	280	14.64	19

F wave

F 波

Annotation	神经	最小 F 反应时间 ms (Minimum F reaction time)	最大 F 反应时间 ms (Maximum F reaction time)	平均 F 反应时间 ms (Mean F reaction time)	% F %	最小 M 反应时间 ms (Minimum F reaction time)	最大 M 反应时间 ms (Maximum M reaction time)	平均 M 反应时间 ms (Mean M reaction time)	最小 M 波幅 mV (Minimum M amplitude)	最大 M 波幅 mV (Maximum M amplitude)	平均 M 波幅 mV (Mean M amplitude)
Left ulnar nerve	L 尺神经 - ADM	47.7	47.7	47.7	10	9.2	21.3	12.1	0.57	1.10	0.85
Right ulnar nerve	R 尺神经 - ADM	26.4	26.4	26.4	10	0.0	0.0	0.0	0.00	0.00	0.00
Right tibial nerve	R 胫神经 - AH	31.7	34.9	33.2	40	14.2	14.5	14.3	0.88	1.20	1.02
Left tibial nerve	L 胫神经 - AH	34.2	34.2	34.2	10	17.0	17.4	17.2	1.43	1.58	1.52
